# Determinants of self-paid rotavirus vaccination status in Kanazawa, Japan, including socioeconomic factors, parents’ perception, and children’s characteristics

**DOI:** 10.1186/s12879-020-05424-6

**Published:** 2020-09-29

**Authors:** Megumi Hara, Rie Koshida, Kaoru Araki, Masahide Kondo, Yoshio Hirota

**Affiliations:** 1 Department of Preventive Medicine, Faculty of Medicine, Saga University, 5-1-1 Nabeshima, Saga, 849-8501 Japan; 2 Health Affairs Department, Kanazawa City, 1-1-1 Hirosaka, Kanazawa, Ishikawa 920-8577 Japan; 3 Department of Pediatrics, Faculty of Medicine, Saga University , 5-1-1 Nabeshima, Saga, 849-8501 Japan; 4 Department of Health Care Policy and Health Economics, Faculty of Medicine, University of Tsukuba, 1-1-1 Tenmoudai, Tsukuba, Ibaraki, 305-8577 Japan; 5 College of Healthcare Management, 960-4, Takayanagi, Setaka-machi, Miyama-shi, Fukuoka, 835-0018 Japan; 6 Clinical Epidemiology Research Center, Medical Co. LTA, 3-5-1 Kashii-Teriha Higashi-ku, Fukuoka, 813-0017 Japan

**Keywords:** Rotavirus vaccine, Self-paid vaccine, Coverage, Socioeconomic status

## Abstract

**Background:**

Japan’s National Immunization Program does not cover rotavirus vaccine and no government subsidies are available. This study aimed to measure the uptake of and determinants that influenced self-paid rotavirus vaccination, including socioeconomic status and relative poverty.

**Methods:**

We conducted a cross-sectional study at health check-ups for all children aged 18 months in Kanazawa, Japan, between December 2017 and July 2018. Community nurses collected information on self-paid vaccination history, parents’ perceptions of and recommendations for rotavirus vaccine, and socioeconomic status in interviews using a unified questionnaire. We used multivariable logistic regression to assess vaccine uptake and possible determinants.

**Results:**

In total, 1282 participants were enrolled. The estimated rotavirus vaccine coverage was 72.9%. Perceptions that rotavirus gastroenteritis was serious and that the rotavirus vaccine was effective, pediatricians’ recommendations, information from the city office, magazine and Internet articles, and higher parental education level were associated with higher rotavirus vaccine uptake. Lower household income was associated with decreased rotavirus vaccine uptake. Vaccine expense, fear of adverse reactions to the vaccine, number of household members and siblings, and children’s characteristics were not correlated with rotavirus vaccination. Poverty was associated with decreased rotavirus vaccine uptake, even after adjustment for other determinants (adjusted odds ratio 0.49, 95% confidence interval: 0.26–0.90).

**Conclusion:**

Parents’ perceptions, socioeconomic status, relative poverty, and pediatricians’ recommendations are determinants of vaccination. This study suggests that appropriate information about rotavirus vaccine, subsidies for those of lower socioeconomic status, and national recommendations are necessary to achieve higher coverage.

## Background

Rotavirus has strong infectivity. It is difficult to prevent infection by hand washing or hygiene, and it is the leading etiology for diarrhea mortality among children aged 5 years and under [1]; therefore, the World Health Organization (WHO) recommended inclusion of rotavirus vaccines in national immunization programs (NIP) in 2009 [2]. Two live oral vaccines are internationally available: a single strain human rotavirus vaccine (Rotarix**®**, GlaxoSmithKline Biologicals, Rixansart, Belgium) [3] and a human-bovine reassortant vaccine (RotaTeq**®**, Merck & Co., Inc., Rahway, NJ, USA) [4]. Four additional rotavirus vaccines are licensed, two in India (Rotavac and Rotasiil), one in China (Lanzhou lamb rotavirus vaccine), and one in Vietnam (Rotavin-M1) [5]. However, rotavirus vaccine is not part of the NIP in many countries, especially in Asia [6].

In the US, there was a well-known risk for intussusception associated with an earlier rotavirus vaccine (RRV-TV, Rotashield, Wyeth Laboratories), which was approved in 1998 and stopped in 1999 [7]. Qualitative research reported that safety concerns and lack of knowledge about rotavirus gastroenteritis (RVGE) were barriers to vaccination when the new rotavirus vaccine (RotaTeq**®**) was approved in 2006 [8]. In Canada, rotavirus vaccination is recommended for all infants, although not all provinces have publicly funded programs. A Canadian study reported that increased knowledge and more positive attitudes toward the vaccine among parents increased the coverage of rotavirus vaccine [9]. Few previous studies have evaluated the influence of parents’ socioeconomic factors on vaccine uptake, although one study reported lower parental socioeconomic status and lower parental concerns about vaccine were determinants of not being vaccinated [10].

In Japan, Rotarix**®** and RotaTeq**®** became available on the private market in November 2011 and July 2012, respectively. Because of the high effectiveness of these vaccines [11, 12] and their impact on reducing hospitalization from RVGE [13–16], rotavirus vaccine will be included in Japan’s NIP in October 2020; however, vaccination is self-paid at present. Additionally, vaccination coverage in Japan is unclear because Japan has no vaccination registry. A previous study in Japan reported that higher annual household income and positive maternal opinion of voluntary vaccination were associated with increased voluntary vaccination uptake, whereas a larger number of siblings was associated with decreased voluntary vaccination uptake [17]. However, that study did not evaluate specific determinants of rotavirus vaccination. As of May 2020, rotavirus, mumps, and influenza vaccines are self-paid vaccines for children in Japan. Newer vaccines, such as rotavirus vaccine, are more expensive compared with those that have been used for many decades, such as the mumps and influenza vaccines. In Japan, the cost of vaccination includes the doctor’s technical fee for administering the vaccine (around US $33 per dose) [18], meaning the cost per full course of rotavirus vaccine is around US $283. This cost is higher than the price per vaccination course elsewhere, with costs ranging from approximately US $0.50 in GAVI-eligible countries to US $185–$226 in the US [19]. Conversely, the costs (including doctor’s technical fee for administration) for the first dose of mumps vaccine and first two doses of influenza vaccine were reported at US $30–$70 and US $40–$80, respectively [20]. The high cost of the rotavirus vaccine may be a barrier to vaccination. However, the impact of socioeconomic factors on rotavirus vaccination uptake has not been evaluated in Japan, especially considering household income.

The impact of household income on self-paid vaccine uptake may differ according to the number of siblings. Therefore, equivalized household income, which is the household income divided by the number of household members, is useful for evaluating economic status and self-paid vaccine uptake. Relative poverty, which is commonly defined as an equivalized household income less than 50% of the national median, is also associated with children’s health, including infant mortality, low birth weight, not having immunizations, child mortality due to unintentional injuries, childhood obesity, and mental health problems [21]. According to the Comprehensive Survey of Living Conditions conducted by Japan’s Ministry of Health, Labour and Welfare, the relative poverty rate increased from 13.5% in 1991 to 15.7% in 2015 [22]. Previous studies reported associations between socioeconomic deprivation and vaccine uptake [23, 24]. Therefore, it is necessary to evaluate the influence of relative poverty on rotavirus vaccine uptake.

This study aimed to measure the uptake of rotavirus vaccine and determinants that influenced self-paid rotavirus vaccination, including socioeconomic factors, relative poverty, parents’ perceptions, and children’s characteristics.

## Methods

### Study design and setting

This cross-sectional study was conducted at the 18-month child health check-up in Kanazawa, Japan, between December 2017 and July 2018. Kanazawa is the largest city in Japan’s Hokuriku region. In 2018, the total population was about 465,000 people and there were around 3800 births. The Japanese government provides a nationwide continuum of maternal, newborn, and child healthcare, the central component of which is the maternal and child health handbook [25]. When pregnant women register, the local government provides them with a maternal and child health handbook; all services then start and follow a standard schedule. Pregnant women bring the handbook to their maternity clinic of choice for antenatal care, and their doctor records antenatal care results for them and their fetus in the handbook. After giving birth, all data regarding childbirth are recorded in the handbook, and mothers receive a birth certificate from the doctor that they take to local government to register the birth. Local governments then provide all registered infants with health information, healthcare advice, and vouchers for child health check-ups and immunizations that are included in the NIP. The main contents of the maternal and child health handbook are described in detail elsewhere [26]. Child health and development check-ups (birth to age 6 years) and immunization status are also recorded in the handbook, regardless of whether they are included in the NIP. Local governments provide health check-ups for all children aged 18 months under the Maternal and Child Health Act. Kanazawa has three welfare and health centers that provide health check-ups for all children aged 18 months. The annual reports of the health and welfare services for children in Kanazawa indicate that the participation rate in health check-ups for children aged 18 months is around 98.9% [27].

 At the time of the children’s health check-up, trained community nurses informed parents about this study and obtained consent for study participation. Then, parents were interviewed using a unified questionnaire that investigated factors that potentially influence rotavirus vaccine uptake. The questionnaire covered parents’ perceptions about RVGE and the rotavirus vaccine, recommendations received or information obtained about rotavirus vaccine, and socioeconomic factors (such as number of household members and siblings, parental age and education level, mothers’ employment). Additionally, data were collected on the child’s birth weight, birth order, underlying diseases (food allergies, atopic dermatitis, nasal allergy, asthma, heart disease, and respiratory disease), daycare service use, and vaccination status for self-paid vaccines (including rotavirus, mumps, and influenza vaccines). Immunization status was ascertained from the maternal and child health handbook, in which all vaccination history must be recorded in Japan as per the Maternal and Child Health Act.

To examine whether relative poverty resulted in a 50% reduction of rotavirus vaccine uptake, a significant effect size of 50% and assuming vaccination uptake of 50%, a percentage of factors associated with vaccination among unvaccinated subjects of 20%, two-sided significance of 0.05, and power of 0.8, this study required 1124 participants.

### Definition and estimation of rotavirus vaccine coverage

Ensuring that vaccination indicators (such as vaccine coverage and uptake) are clearly and consistently defined is important for effective communication of outcomes [28]. According to the US Centers for Disease Control and Prevention, childhood vaccination coverage is defined as the percentage of children in the target population who received a dose of a recommended vaccine [29]. Vaccine uptake is most commonly defined as the absolute number of people who received a specific dose; that is, the numerator in the vaccine coverage calculation. In Japan, rotavirus vaccine is recommended to start at 2 months of age and should be completed before 6 months; therefore, all children in this study had passed the recommended age range. We defined vaccine uptake as the number of children that received at least one dose of rotavirus vaccine. To estimate vaccination coverage, we calculated the proportion of self-reported rotavirus vaccine uptake and that of record-confirmed rotavirus vaccine uptake among children whose parents participated in this survey.

### Evaluating equivalent household income and relative poverty rate

We recorded annual household income using five categories: less than \2,000,000; \2,000,000–\3,999,999; \4,000,000–\5,999,999; \6,000,000 or more; and unknown. To estimate the mid-point of an open-ended income category, we used formulas described by Parker et al. [30] These formulas are based on Pareto’s law of income distribution, which states that the logarithm of the percentage of units with an income in excess of a certain value is a negatively sloped linear function of the logarithm of that value. According to this theory [30], Celeste et al. defined the median income for the top-coded category = 10^(0.301/v)*(X), where X = lower value of the top-coded/open-ended category and v = c − d/b − a. Where a = the log of the lower limit of the interval preceding the top-coded/open-ended category; b = log of the lower limit of the top-coded/open-ended category; c = the log of the sum of the frequencies in the top-coded category and the category preceding it; and d = the log of the frequencies in the top-coded category [31].

We equivalized household income using the square root of household scale, which means that household income was divided by the square root of household size [32]. For example, a household of four persons has needs twice as large as one composed of single person. The relative poverty rate was defined as the proportion of children with an equivalent household income less than 50% of the median of all surveyed children, following the definition of the Organisation for Economic Co-operation and Development (OECD) [33].

### Statistical analysis

We first assessed correlations in parents’ perception of rotavirus vaccine and RVGE, socioeconomic factors, and children’s characteristics between vaccinated and unvaccinated children. Odds ratios (OR) and 95% confidence intervals (CI) were calculated using a univariate logistic regression model. Next, multiple logistic regression analysis was performed to investigate correlations between rotavirus vaccination status and potentially influential factors. Because birth order, number of siblings, and number of household members are highly correlated, we only included the number of siblings in the multivariable model. In Model 1, we adjusted for siblings (≥2 or < 2 [reference]), mother’s age (< 30 years or ≥ 30 years [reference]), parental educational level of university or above (yes/no [reference]), and household income below \4,000,000 (yes/no [reference]). In Model 2, we further adjusted for perceptions of RVGE, vaccine effectiveness, pediatrician recommendation, and information from city papers, magazines, and the Internet. For the equivalent income and relative poverty rate analyses, we excluded cases where data for household income and household size were not available, and performed additional analyses of the association between relative poverty status and self-paid vaccine uptake using complete data. Median household equivalent income was compared by rotavirus vaccine uptake status using the Mann–Whitney U-test. We evaluated associations between relative poverty and rotavirus vaccine uptake and uptake of other self-paid vaccines using multiple logistic regression analysis with adjustment for possible confounders. Parents’ education level was entered into Model 1. Model 2 was further adjusted for parents’ perceptions, recommendation, and information, which were significantly correlated with rotavirus vaccine uptake. All analyses were conducted using SAS version 9.4 (SAS Inc., Cary, NC).

## Results

In total, 1303 children attended the 18-month health check-up, and data for 1282 children were gathered (participation rate 98.4%). Of these children, 664 (52.0%) were boys. Table 1 shows parents’ perceptions and factors possibly related to rotavirus vaccine uptake. We found that 73% of parents believed RVGE was a serious disease, 66% believed rotavirus vaccine was effective, 36% worried about adverse reactions to the rotavirus vaccine, and most (90%) thought that the rotavirus vaccine was expensive. Parents were more likely to have received a recommendation from a pediatrician to vaccinate their child for rotavirus than from an obstetric doctor. Parents were more likely to get information from city papers than from magazines or the Internet. An analysis of socioeconomic factors showed that about 60% of children lived in households with four or more members and two siblings. More fathers than mothers had a university-level education. About 70% of mothers were employed, and about 40% of families had an annual income lower than \4,000,000. About 10% of children were born weighing less than 2500 g, about 30% had any primary diseases, and about 60% used daycare services.

**Table 1:** Parents’ perceptions and factors potentially related to rotavirus vaccine uptake (*n* = 1282)

	Agree ***n***, (%)	Disagree ***n***, (%)	Uncertain ***n***, (%)
***Perception***			
I believe that RVGE is a serious disease	936 (73.0)	89 (6.9)	257 (20.0)
I believe that the rotavirus vaccination is effective	846 (66.0)	45 (3.5)	391 (30.5)
I worry about adverse reactions to the rotavirus vaccine	455 (35.5)	601 (46.9)	226 (17.6)
I think that the rotavirus vaccine is expensive	1149 (89.6)	122 (9.5)	11 (0.9)
	**Yes** ***n*, (%)**	**No** ***n*, (%)**	**Missing** ***n*, (%)**
***Recommendation***			
Obstetric doctor recommended the rotavirus vaccine	125 (9.8)	1150 (89.7)	7 (0.5)
Pediatrician recommended the rotavirus vaccine	579 (45.2)	696 (54.3)	7 (0.5)
I read information about rotavirus vaccine in a city paper	838 (65.4)	436 (34.0)	8 (0.6)
I read information about rotavirus vaccine in magazines and on the Internet	478 (37.3)	797 (62.2)	7 (0.5)
***Socioeconomic status***			
Household members ≥4	765 (59.7)	517 (40.3)	
Siblings ≥2	712 (55.5)	570 (44.5)	
Father’s age < 30 years	214 (16.7)	1033 (80.6)	35 (2.7)
Mother’s age < 30 years	290 (22.6)	987 (77.0)	5 (0.4)
Father’s education level ≥ university	700 (54.6)	582 (45.4)	
Mother’s education level ≥ university	454 (35.4)	828 (64.6)	
Mother has job	878 (68.5)	404 (31.5)	
Household income below \4,000,000	470 (36.7)	812 (63.3)	
***Children’s characteristics***			
Birth weight < 2500 g	116 (9.0)	1166 (91.0)	
First child	601 (46.7)	675 (52.6)	6 (0.5)
Have primary diseases	360 (28.1)	871 (67.9)	51 (4.0)
Daycare use	714 (55.6)	529 (41.3)	39 (3.0)
***Self-paid vaccine uptake***			
Rotavirus vaccine	921 (71.8)	347 (27.1)	14 (1.1)
Mumps vaccine	679 (53.0)	587 (45.8)	16 (1.2)
Influenza vaccine	645 (50.3)	629 (49.1)	8 (0.6)

*RVGE* rotavirus gastroenteritis

Among the 1282 participating children, 935 had received at least one dose of rotavirus vaccine, giving a proportion of self-reported rotavirus vaccine uptake of 72.9%. However, the vaccination status for 14 children (1.5%) was not recorded in their maternal and child health handbook. After excluding these 14 children, the record-confirmed rotavirus vaccine uptake was 921 (of 1268 children), giving a proportion of record-confirmed rotavirus vaccine uptake of 72.6%. Among these 921 children, 565 (61.3%) were vaccinated with Rotarix®; 561 were fully vaccinated and 4 received only one dose. Similarly, of the 356 (38.7%) children that were vaccinated with RotaTeq®, 351 were fully vaccinated and five children received two doses. Regarding other self-paid vaccines, 669 children received the mumps vaccine and 625 received the influenza vaccine, meaning that the proportions of record-confirmed vaccine uptake were 52.2 and 49.3% for mumps and influenza, respectively.

Table 2 shows determinants influencing rotavirus vaccine uptake. In the crude model, parents’ belief that RVGE was serious and that the vaccine was effective, pediatricians’ recommendation, parental education level, information about rotavirus vaccine from city papers/magazines/the Internet, and the first child were associated with higher rotavirus vaccine uptake. Larger numbers of household members and siblings, mother’s age < 30 years and lower household income were associated with decreased rotavirus vaccine uptake. In the multivariate model, parents’ belief that RVGE was serious and vaccination was effective, pediatricians’ recommendation, information about rotavirus vaccine from city papers, and mother’s higher education level were associated with increased vaccine uptake. However, lower household income was associated with decreased vaccine uptake (adjusted OR: 0.66; 95%CI: 0.49–0.90). Regarding determinants influencing the self-paid uptake of other vaccines, father’s higher educational level was correlated with increased uptake of both mumps and influenza vaccines, whereas larger number of siblings was associated with decreased uptake of these vaccines. A lower household income and mother’s age < 30 years were associated with decreased mumps vaccine uptake; the adjusted ORs for lower household income and mother’s age < 30 years were 0.67 (95%CI: 0.52–0.87) and 0.60 (95%CI: 0.44–0.81), respectively. However, these factors were not associated with influenza vaccine uptake. (Additional File 1).

**Table 2:** Parents’ perceptions of rotavirus vaccine, rotavirus gastroenteritis, recommendation for vaccination, socioeconomic status, and children’s characteristics by rotavirus vaccine uptake (*n* = 1268)

	Vaccinated (*n* = 921)	Unvaccinated (n = 347)	Crude OR (95% CI)	Model 1 aOR^a^ (95% CI)	Model 2 aOR^b^ (95% CI)
***Perception***					
I believe that RVGE is a serious disease	738 (80.1%)	184 (53.0%)	**3.59 (2.75–4.69)**	**N/A**	**2.75 (2.01–3.76)**
I believe that the rotavirus vaccination is effective	720 (78.2%)	115 (33.1%)	**7.27 (5.53–9.56)**	**N/A**	**5.66 (4.20–7.63)**
I worry about adverse reactions to the rotavirus vaccine	333 (36.2%)	116 (33.4%)	1.12 (0.87–1.46)	**N/A**	
I think that the rotavirus vaccine is expensive	829 (90.0%)	309 (89.1%)	1.06 (0.70–1.61)	**N/A**	
***Recommendation***					
Obstetric doctor recommended the rotavirus vaccine	99 (10.8%)	25 (7.2%)	1.55 (0.98–2.45)	**N/A**	
Pediatrician recommended the rotavirus vaccine	462 (50.2%)	108 (31.1%)	**2.23 (1.72–2.90)**	**N/A**	**2.04 (1.50–2.78)**
I read information about rotavirus vaccine in a city papers	642 (69.7%)	185 (53.3%)	**1.99 (1.55–2.57)**	**N/A**	**1.77 (1.30–2.41)**
I read information about rotavirus vaccine in magazines and on the Internet	382 (41.5%)	90 (26.0%)	**2.03 (1.54–2.67)**	**N/A**	1.3 (0.96–1.83)
**Socioeconomic status**					
Household members ≥4	449 (54.2%)	258 (74.4%)	**0.41 (0.31–0.54)**		
Siblings ≥2	452 (49.1%)	252 (72.6%)	**0.36 (0.28–0.48)**	**0.30 (0.22–0.41)**	**0.36 (0.26–0.49)**
Father’s age < 30 years	142 (15.8%)	68 (20.5%)	0.73 (0.53–1.00)		
Mother’s age < 30 years	192 (21.0%)	95 (27.4%)	**0.70 (0.53–0.94)**	0.73 (0.49–1.09)	
Father’s education level ≥ university	541 (58.7%)	151 (43.5%)	**1.85 (1.44–2.37)**	1.28 (0.93–1.65)	
Mother’s education level ≥ university	377 (40.9%)	72 (20.8%)	**2.65 (1.98–3.54)**	**2.00 (1.44–2.78)**	**2.27 (1.61–3.20)**
Mother’s employment	631 (68.5%)	237 (68.3%)	1.01 (0.77–1.32)		
Household income below \4,000,000	301 (32.7%)	162 (46.7%)	**0.55 (0.43–0.71)**	**0.61 (0.46–0.80)**	**0.66 (0.49–0.90)**
***Children’s characteristics***					
Birth weight < 2500 g	82 (8.9%)	31 (8.9%)	1.00 (0.65–1.54)		
First child	488 (53.2%)	106 (30.7%)	**2.57 (1.97–3.34)**		
Having primary diseases	264 (29.9%)	92 (27.6%)	1.12 (0.84–1.48)		
Daycare use	518 (57.9%)	187 (56.0%)	1.08 (0.84–1.39)		

*CI* confidence interval, *OR* odds ratio, *aOR* adjusted odds ratio, *RVGE* rotavirus gastroenteritis

^a^ Adjusted for siblings (≥2 or < 2 [reference]), mother’s age (< 30 years or ≥ 30 [reference]), father’s educational level ≥ university (yes/no [reference]), mother’s educational level ≥ university (yes/no [reference]), and household income below \4,000,000 (yes/no [reference]).

^b^ Further adjusted for perceptions of RVGE, vaccine effectiveness, pediatrician’s recommendation, and information from city papers, magazines, and the Internet

Table 3 lists reasons for parents not vaccinating their children for rotavirus (*n* = 347). The most common reason was “Rotavirus vaccine is not included in the NIP” (*n* = 129, 37.3%), followed by “Parents have to pay for uptake” (n = 129, 30.4%). Among 105 parents who said they did not vaccinate because of the cost, 44 (41.9%) parents might have vaccinated if the vaccine was free and 52 (49.5%) might have vaccinated if it was less than \5000 (Additional File 2).

**Table 3:** Reasons for not vaccinating children (*n* = 347)

Rotavirus vaccine is not included national immunization program	129	(37.3%)
Parents have to pay up to \30,000 (approximately US $300) for uptake	105	(30.4%)
Suspicious about the vaccine’s effectiveness	20	(5.8%)
Worry about adverse reactions	15	(4.3%)
Nobody recommended	14	(4.1%)
Missed the opportunity	11	(3.2%)
I don’t think that RVGE is serious	11	(3.2%)
I did not know about the rotavirus vaccine	8	(2.3%)
Rotavirus vaccine is not necessary, because our child does not use daycare.	6	(1.7%)
Others	28	(8.1%)

*RVGE* rotavirus gastroenteritis

Information about household income was available for 991 of the 1282 participants. The estimated mid-point income for the upper open-ended category was \9,272,912 (Additional File 3). After excluding participants with missing data for number of household members and siblings, we used data for 990 participants to calculate household equivalent income and relative poverty rate. The median household equivalent income (using the per square root scale) was \2,886,751. The median household equivalent income was significantly higher among those who were vaccinated (\2,886,571) than those who were unvaccinated (\2500,00) (*P* < 0.05). The poverty line, which was half of the median household equivalent income, was estimated as \1,443,375; 65 participants (6.6%) were defined as having relative poverty.

Table 4 shows relative poverty and self-paid vaccine uptake status using complete data. Relative poverty status was associated with decreased rotavirus vaccine uptake, even after adjusting for parents’ education level and other possible influencing factors (adjusted OR: 0.49, 95%CI: 0.26–0.90). Relative poverty was also associated with decreased mumps vaccine uptake (crude OR: 0.49, 95%CI: 0.29–0.81), although this correlation was not significant after adjusting for parental educational level. Relative poverty was not correlated with influenza vaccine uptake.

**Table 4:** Relative poverty status and self-paid vaccine uptake (*n* = 990)

	Vaccinated	Unvaccinated	Crude OR (95% CI)	Model 1^a^ aOR (95% CI)	Model 2^b^ aOR (95% CI)
Rotavirus vaccine					
Not poverty	700	225	1.00 (reference)	1.00 (reference)	1.00 (reference)
Poverty	35	30	**0.38 (0.23–0.62)**	**0.48 (0.29–0.81)**	**0.49 (0.26–0.90)**
Mumps virus vaccine					
Not poverty	521	404	1.00 (reference)	1.00 (reference)	
Poverty	25	40	**0.49 (0.29–0.81)**	0.60 (0.35–1.02)	
Influenza vaccine					
Not poverty	470	455	1.00 (reference)	1.00 (reference)	
Poverty	26	39	0.65 (0.40–1.13)	0.80 (0.47–1.35)	

*OR* odds ratio, *aOR* adjusted odds ratio, *CI* confidence interval

^a^ Adjusted for parents’ education level ≥ university (yes/no [reference])

^b^ Further adjusted for perceptions of RVGE, vaccine effectiveness, pediatrician’s recommendation, and information from city papers, magazines, and the Internet

## Discussion

To our knowledge, this was the first study from Asia to assess self-paid rotavirus vaccine uptake status and determinants of vaccine uptake. Although the rotavirus vaccine is currently not included in the NIP and its cost (including doctors’ technical fee) for full course vaccination is considered expensive in Japan, vaccine coverage in Kanazawa was estimated at 72.9%. Rotavirus vaccine uptake was associated with parental belief that RVGE was serious and vaccination was effective, recommendation from a pediatrician, information about rotavirus vaccine from city papers, and higher mother’s age and higher education level. However, lower household income and relative poverty were associated with decreased rotavirus vaccine uptake, even after adjusting for other determinants.

In terms of the influence of socioeconomic factors on rotavirus vaccination, ecological studies suggest that the most deprived areas have the lowest coverage and higher rates of hospitalization for acute gastroenteritis caused by rotavirus [24, 34, 35]. However, ecological studies were not able to evaluate socioeconomic factors and vaccine uptake with consideration of the influence of parental perceptions, recommendations received, and other individual-level determinants. Although some cross-sectional studies evaluated the influence of socioeconomic factors and other determinants on rotavirus vaccine uptake [10, 36, 37], few studies evaluated household income and vaccine uptake [10, 36]. MacDonald et al. conducted a retrospective population-based cohort study of Canadian children born between January 1, 2008 and December 31, 2013, and found that income had a strong influence in urban areas, but not in rural regions where coverage was lower overall [37]. Our results support the idea that household income influences self-paid vaccination uptake in areas where vaccination coverage is high, even after adjusting for other determinants.

The pivotal point of this study was that we evaluated relative poverty and self-paid vaccine uptake. Relative poverty is reported to be significantly associated with child health and wellbeing [21]. According to the Comprehensive Survey of Living Conditions conducted by Japan’s Ministry of Health, Labour and Welfare, the relative poverty rate of children in 2015 was 13.9%, which was 14th highest among 35 OECD countries [22]. Therefore, evaluations of relative poverty and child health-related issues in Japan are important. To our knowledge, this is the first study to show that relative poverty was associated with decreased rotavirus vaccine uptake. Conversely, the uptake of other self-paid vaccines (mumps and influenza) was not correlated with relative poverty. The higher cost of rotavirus vaccine compared with other self-paid vaccines may explain this discrepancy. About one-third of parents of unvaccinated children said they did not get the rotavirus vaccine for their children because of the cost. Therefore, a rotavirus vaccination subsidy for children with relative poverty or universal vaccination may improve vaccine uptake. Fortunately, rotavirus vaccine will be included in the NIP in Japan from October 2020, meaning that the influence of relative poverty will no longer be a reason for non-vaccination.

Consistent with previous reports [9, 10, 36–39], rotavirus vaccine uptake was positively associated with parental awareness of RVGE severity and confidence in rotavirus vaccine effectiveness, mother’s age and educational level. In contrast to a previous report, we found that awareness of safety was not associated with uptake [40]. Regarding vaccine safety, less than 40% of parents worried about adverse reactions to rotavirus vaccine, although there is a known small risk for intussusception after first dose vaccine [41]. This may indicate that parental decisions regarding vaccination may depend on their knowledge and information about RVGE and rotavirus vaccine, including effectiveness and safety. In this study, recommendation from a pediatrician and information from city papers were associated with rotavirus vaccine uptake. Because childhood vaccines are administered at pediatric clinics in Japan, the role of pediatricians in providing information and recommendation for rotavirus vaccine is important to achieve high coverage.

This study had some limitations. First, we conducted this study in an urban area of Japan and did not include a rural area; therefore, study participants are unlikely to be representative of all Japanese children. The relative poverty rate in this study was 6.6%, which was lower than that for all of Japan (15.7%) in 2016. The low relative poverty rate in this area might have influenced vaccine coverage and determinants. Additionally, convenience of access to vaccination may differ between urban and rural areas. Geographical accessibility is a factor that influences vaccine hesitancy [42]. Further studies in rural areas are needed to clarify this point. Second, there is potential for recall bias in parents’ perceptions and receiving recommendation because those items were measured after vaccination. However, we ascertained vaccination status from the maternal and child health handbook, and questions relating to current socioeconomic factors were answered. Therefore, we consider that the correlation between self-paid vaccine uptake and socioeconomic factors was not affected by recall bias.

## Conclusions

This study revealed that parental socioeconomic status, especially relative poverty, was associated with decreased uptake of self-paid rotavirus vaccine in Japan, which is a high-income country with high vaccination coverage. Increasing vaccination coverage requires inclusion of rotavirus vaccine in the NIP or provision of a subsidy for children in relative poverty. Additionally, recommendation for and information about rotavirus vaccine from pediatricians or city offices is necessary to positively influence parents’ vaccination decisions, even after introducing rotavirus vaccine into the NIP.

## Supplementary information

**Additional file 1.** Socioeconomic status and characteristics of children by self-paid vaccine uptake

**Additional file 2.** Affordable cost of a full vaccination course among parents who did not vaccinate their children because of the high cost

**Additional file 3.** Household income categories and their medians

## Data Availability

The datasets used and/or analysed during the current study are available from the corresponding author on reasonable request.

## Abbreviations

CI: Confidence interval
NIP: National Immunization Program
OECD: Organisation for Economic Co-operation and Development
OR: Odds ratio
RVGE: Rotavirus gastroenteritis
WHO: World Health Organization
