# Supplementary material for: Determinants of self-paid rotavirus vaccination status in Kanazawa, Japan, including socioeconomic factors, parents’ perception, and children’s characteristics
Source: BMC Infect Dis. 2020 Sep 29;20:712. doi: 10.1186/s12879-020-05424-6 (PMC7526161; doi:10.1186/s12879-020-05424-6)
Supplement: Supplementary file 1 — Additional file 1. Socioeconomic status and characteristics of children by self-paid vaccine uptake [file 12879_2020_5424_MOESM1_ESM.docx]

**Additional file 1.** Socioeconomic status and characteristics of children by self-paid vaccine uptake (n=1,282)

|  | Mumps vaccine uptake | |  | Influenza vaccine uptake | |
| --- | --- | --- | --- | --- | --- |
|  | Crude OR  (95% CI) | aOR^a^  (95% CI) |  | Crude OR  (95% CI) | aOR^b^  (95% CI) |
| **Socioeconomic status** |  |  |  |  |  |
| Household members ≥4 | **0.47 (0.37-0.59)** |  |  | 0.82 (0.66–1.03) |  |
| Siblings ≥2 | **0.40 (0.32–0.50)** | **0.35 (0.83–0.45)** |  | **0.79 (0.63–0.98)** | **0.78 (0.61–0.98)** |
| Father’s age <30 years | 0.92 (0.69–1.24) |  |  | **0.59 (0.44–0.80)** | 0.69 (0.47–1.02) |
| Mother’s age <30 years | **0.66 (0.51–0.86)** | **0.60 (0.44–0.81)** |  | **0.68 (0.52–0.88)** | 0.83 (0.59–1.18) |
| Father’s education level ≥university | **2.01 (1.60–2.51)** | **1.64 (1.27–2.13)** |  | **1.77 (1.41–2.21)** | **1.55 (1.21–1.99)** |
| Mother’s education level ≥university | **1.81 (1.43–2.29)** | 1.27 (0.97–1.67) |  | **1.56 (1.24–1.97)** | 1.28 (0.99–1.65) |
| Mother’s employment | **0.74 (0.58–0.94)** |  |  | 1.22 (0.96–1.54) |  |
| Household income below \4,000,000 | **0.67 (0.53–0.84)** | **0.67 (0.52–0.87)** |  | 0.92 (0.73–1.15) |  |
| ***Children’s characteristics*** |  |  |  |  |  |
| Birth weight <2500 g | 1.20 (0.82–1.77) |  |  | 1.05 (0.72–1.54) |  |
| First child | **2.34 (1.86–2.94)** |  |  | 1.21 (0.97–1.51) |  |
| Having primary diseases | 0.95 (0.74–1.21) |  |  | 0.95 (0.75–1.22) |  |
| Daycare use | **0.79 (0.63–0.99)** | 0.88 (0.70–1.13) |  | 1.22 (0.97–1.52) |  |

CI; confidence interval, OR; odds ratio; aOR, adjusted odds ratio.

^a^ Adjusted for siblings (≥2 or <2 [reference]), mother’s age (<30 years or ≥30 [reference]), father’s educational level ≥university (yes/no [reference]), mother’s educational level ≥university (yes/no [reference]), household income below \4,000,000 (yes/no [reference]), and daycare use (yes/no [reference]).
^b^ Adjusted for siblings (≥2 or <2 [reference]), father’s age (<30 years or ≥30 [reference]), mother’s age (<30 years or ≥30 [reference]), father’s educational level ≥university (yes/no [reference]), and mother’s educational level ≥university (yes/no [reference]).
