# Supplementary material for: Determinants of self-paid rotavirus vaccination status in Kanazawa, Japan, including socioeconomic factors, parents’ perception, and children’s characteristics
Source: BMC Infect Dis. 2020 Sep 29;20:712. doi: 10.1186/s12879-020-05424-6 (PMC7526161; doi:10.1186/s12879-020-05424-6)
Supplement: Supplementary file 2 — Additional file 2. Affordable cost of a full vaccination course among parents who did not vaccinate their children because of the high cost [file 12879_2020_5424_MOESM2_ESM.docx]

**Additional file 2.** Affordable cost of a full vaccination course among parents who did not vaccinate their children because of the high cost. (n=105）

| Free | 44 | (41.9%) |
| --- | --- | --- |
| Less than \5,000 | 52 | (49.5%) |
| Less than \10,000 | 9 | (8.6%) |
| Less than \20,000 | 0 | (0%) |
