# Supplementary material for: Determinants of self-paid rotavirus vaccination status in Kanazawa, Japan, including socioeconomic factors, parents’ perception, and children’s characteristics
Source: BMC Infect Dis. 2020 Sep 29;20:712. doi: 10.1186/s12879-020-05424-6 (PMC7526161; doi:10.1186/s12879-020-05424-6)
Supplement: Supplementary file 3 — Additional file 3. Household income categories and their medians [file 12879_2020_5424_MOESM3_ESM.docx]

**Additional file 3.** Household income categories and their medians (n=991)

| Household income | n | (%) | median |
| --- | --- | --- | --- |
| Less than \2,000,000 | 17 | 1.7 | ¥1,000,000 |
| \2,000,000–3,999,999 | 162 | 16.3 | ¥3,000,000 |
| \4,000,000–5,999,999 | 386 | 39.0 | ¥5,000,000 |
| equal to or above \6,000,000 | 426 | 43.0 | ¥9,272,912 |
